# Recurrent Extraneural Metastatic Medulloblastoma in an Adult Presenting With a Superscan and Treated With Radium-223

**DOI:** 10.7759/cureus.34732

**Published:** 2023-02-07

**Authors:** Benjamin Mou, Ella Mae Cruz-Lim

**Affiliations:** 1 Department of Radiation Oncology, BC Cancer, Kelowna, CAN

**Keywords:** superscan, extraneural, bone metastases, radionuclide therapy, radium-223, metastatic medulloblastoma, adult medulloblastoma

## Abstract

A 32-year-old man with medulloblastoma was initially treated with subtotal resection and craniospinal irradiation. He developed recurrent metastatic disease three years later with extensive bone-only metastases. Biopsy of the bone lesions confirmed metastatic medulloblastoma and restaging investigations demonstrated a superscan with no evidence of recurrence in the craniospinal axis. Extraneural metastatic medulloblastoma is rare, and the presentation with diffuse bone-only metastases with a superscan on imaging is unique. The patient had diffusely painful bone metastases requiring multiple hospitalizations for poor pain control. He declined chemotherapy and was treated with radium-223, an alpha particle emitting radionuclide therapy typically used in metastatic castrate-resistant prostate cancer. The patient received three out of a planned six cycles of radium-223 before it was discontinued due to myelosuppression requiring multiple blood transfusions, and restaging demonstrated local recurrence in the posterior fossa. This is the first report to our knowledge describing the use of radium-223 in a patient with extraneural bone-only metastatic medulloblastoma. Further research into the effect of radium-223 in patients with diffuse bone-only metastases from non-prostate cancer primary tumors is warranted.

## Introduction

Extraneural metastasis is an uncommon presentation of recurrent medulloblastoma affecting 5% to 10% of all medulloblastoma cases [1]. Moreover, adult medulloblastoma is extremely rare, with an incidence of 0.6 per million people annually [2,3]. This case report describes the presentation and management of an adult with medulloblastoma who recurred three years after initial treatment with extensive bone-only metastases presenting with a superscan on imaging and treated with radium-223 therapy. The patient provided verbal and written consent for the details of his case to be published as a case report and institutional research ethics board approval was waived.

## Case presentation

**Figure 1:**
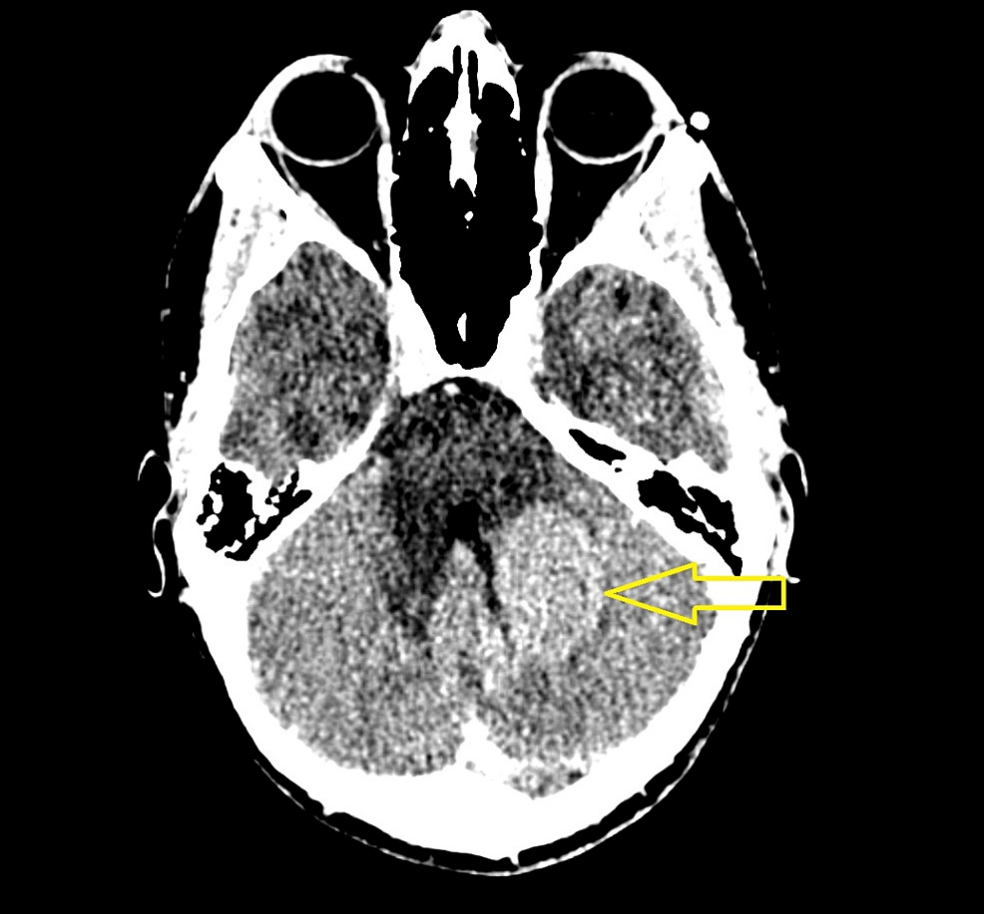
Contrast-enhanced axial CT scan demonstrating a mass in the left cerebellum. CT, computed tomography

**Figure 2:**
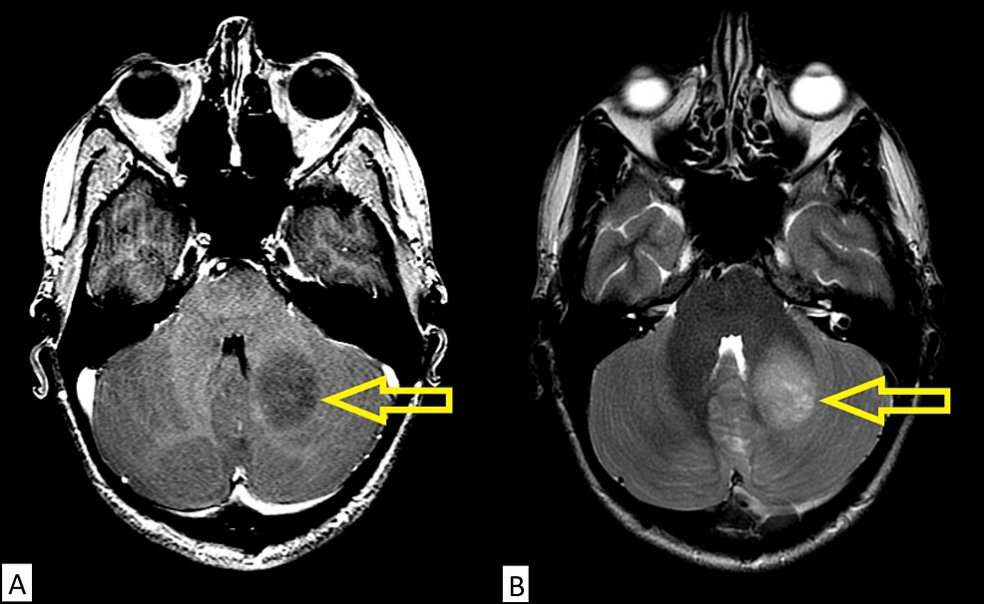
Preoperative MRI: (A) Gadolinium-enhanced T1-weighted and (B) T2-weighted axial images demonstrating a non-enhancing lobulated mass in the left cerebellum. MRI, magnetic resonance imaging

**Figure 3:**
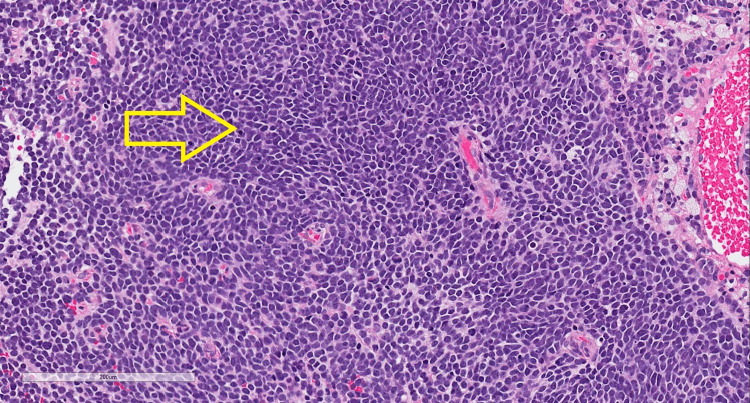
High-power microscopy image of medulloblastoma demonstrating small, round blue cells with diffuse tumor growth pattern.

**Figure 4:**
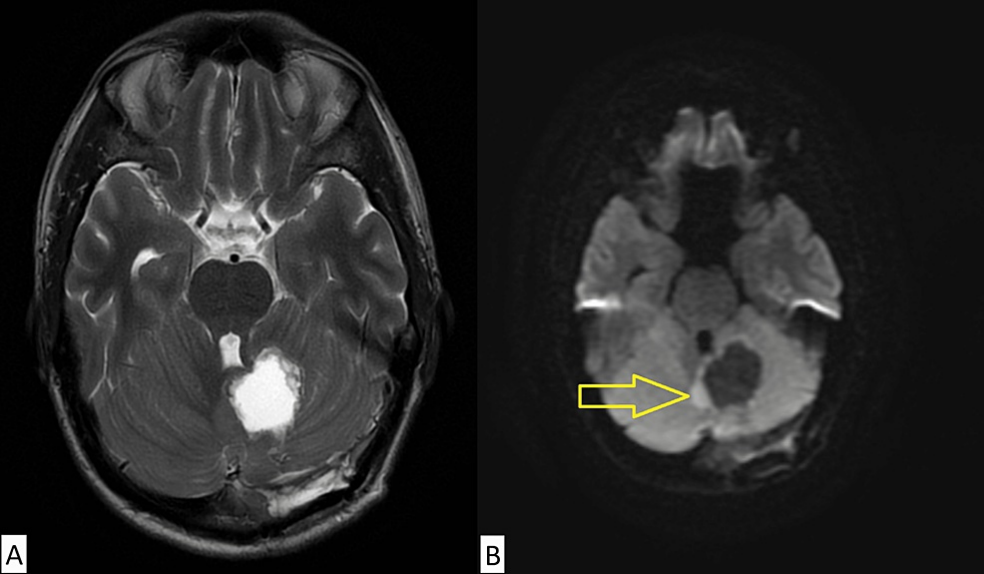
Postoperative MRI: (A) T2-weighted and (B) diffusion-weighted axial images demonstrating residual tumor in the anterior and medial resection cavity. MRI, magnetic resonance imaging

A 32-year-old male presented with symptoms of progressive blurred vision and headaches for several months. Initial assessment by an ophthalmologist demonstrated papilledema, and an urgent computed tomography (CT) scan demonstrated an intra-axial mass in the left cerebellum concerning malignancy (Figure 1). Magnetic resonance imaging (MRI) of the head demonstrated a 3.4 cm × 2.8 cm × 2.2 cm lobulated non-enhancing mass in the left cerebellum involving the inferior portion of the middle cerebellar peduncle and extending across the vermis (Figure 2). The patient underwent left posterior craniectomy, resection of the tumor, and insertion of a right ventricular drain. Pathology demonstrated small, round blue cells with dense cellularity, a sheet-like growth pattern, scattered apoptotic bodies, and brisk mitotic activity (Figure 3). Immunohistochemical staining was positive for synaptophysin, chromogranin, and glial fibrillary acidic protein in an intracytoplasmic distribution. The Ki-67 immunohistochemical study showed a high proliferative index, with approximately 50% of tumor cells positive. Staining for cytokeratin AE1/2, cytokeratin 20, and thyroid transcription factor 1 (TTF-1) was all negative. The final diagnosis was World Health Organization grade 4 medulloblastoma, sonic hedgehog (SHH) subtype, and this was confirmed by a neuropathologist. Postoperative MRI revealed a rim of restricted diffusion along the anterior and medial margins of the left cerebellar tumor resection cavity consistent with residual tumor, with a vermian focus measuring 8 mm × 15 mm (Figure 4). An MRI of the entire spine and lumbar puncture was negative for leptomeningeal dissemination. He was treated postoperatively with external beam radiotherapy using volumetric modulated arc therapy. He was prescribed a dose of 36 Gy in 20 fractions to the craniospinal axis, immediately followed by a boost to the tumor bed to a dose of 18 Gy in 10 fractions for a total prescribed dose of 54 Gy in 30 fractions. The patient had compliance issues with failing to answer phone calls for medical appointments despite numerous attempts to improve communication. He failed to attend his last fraction of radiotherapy. Consolidative chemotherapy was recommended after radiotherapy, but the patient declined due to concerns about toxicity.

**Figure 5:**
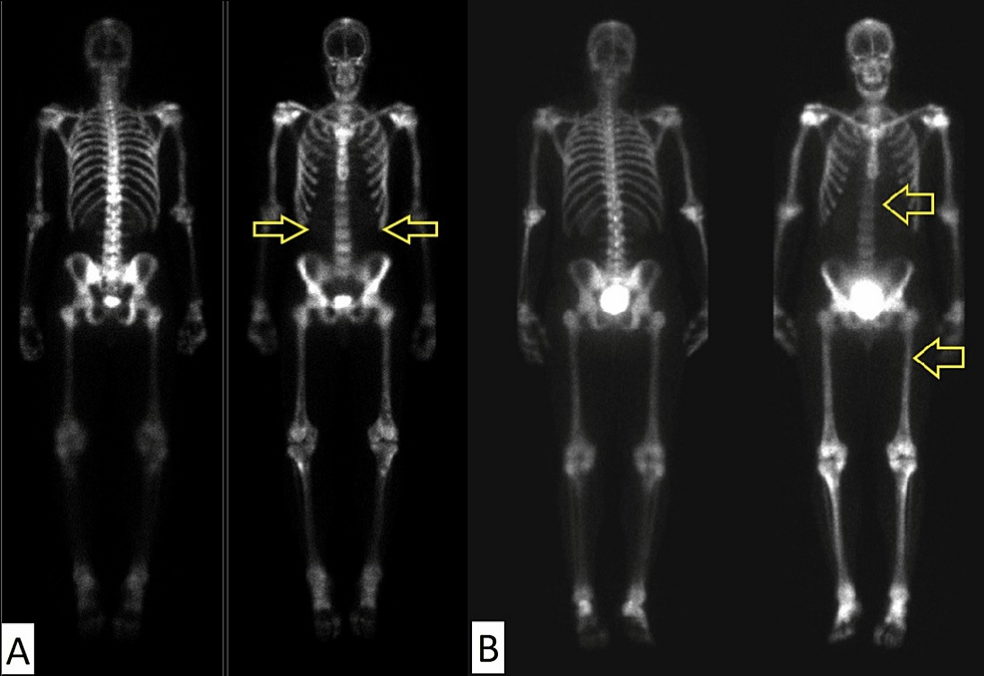
(A) A superscan demonstrating diffuse heterogeneously increased activity in the axial and appendicular skeleton with associated soft tissue and renal suppression; (B) a restaging bone scan demonstrating a persistent superscan; however, with the heterogeneity of bone remodeling in the lower thoracic spine and distal left femur consistent with disease improvement.

Posttreatment MRI of the brain and spine demonstrated a complete radiologic response with complete resolution of the previously seen residual tumor. Subsequent surveillance imaging was performed periodically but was challenging due to poor patient compliance, with multiple missed appointments over the years following his initial treatment, which were later compounded by the COVID-19 pandemic. Three years after the patient’s initial diagnosis and treatment, he presented to the emergency department in a pain crisis. His workup included X-rays, CTs, and bone scans. The bone scan demonstrated diffuse osseous metastatic disease throughout the axial and appendicular skeleton with loss of soft tissue and renal uptake consistent with a superscan (Figure 5A). Restaging MRI of the head and spine did not demonstrate any evidence of local recurrence or leptomeningeal spread. Body CT did not identify any extraneural soft tissue metastases. A biopsy of a sclerotic lesion in the pelvis demonstrated the same histopathologic features and immunohistochemical staining profile as the original pathology specimen in keeping with a diagnosis of metastatic medulloblastoma. Additional molecular genetics testing demonstrated a missense variant in exon 20 of PIK3CA. The patient was hospitalized twice for pain management due to poorly controlled pain from his bone metastases despite opioid analgesic therapy.

**Figure 6:**
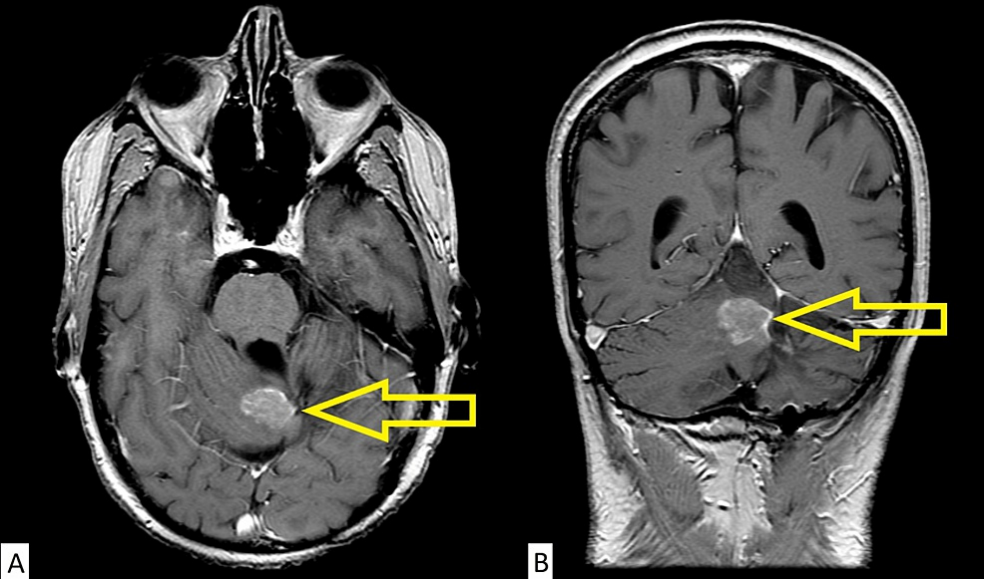
Restaging MRI. Gadolinium-enhanced T1-weighted (A) axial and (B) coronal images of the brain demonstrating local recurrence within the vermis. MRI, magnetic resonance imaging

Following discussion at the multidisciplinary neuro-oncology tumor board conference, palliative chemotherapy with temozolomide and etoposide was recommended; however, in light of the patient’s history of refusing chemotherapy, bone-directed radionuclide therapy was also recommended as an alternative treatment option as the patient’s recurrence was limited to the bone with no soft tissue metastases. Palliative radiotherapy with further external beam radiotherapy was not favored due to the previous course of craniospinal irradiation and the extensive distribution of the patient’s diffusely symptomatic skeletal metastases. Radionuclide therapy with radium-223 was recommended based on the patient’s symptomatic and radiologic disease presentation, extrapolating from its use in metastatic prostate cancer where superscans are more common. Radium-223 was favored over other radionuclide options, due to the local availability of radium-223 and its more favorable toxicity profile with less risk of myelosuppression compared to beta particle-emitting radionuclides. The patient declined chemotherapy due to concerns about toxicity but consented to radium-223, which was approved for use in this patient following an application through the provincial compassionate access program. The patient was treated with 55 kBq/kg delivered intravenously every four weeks using the same protocol for patients with metastatic castrate-resistant prostate cancer (mCRPC) [4]. The patient required multiple blood transfusions before each cycle of radium-223 due to anemia. He only received three out of a planned six cycles of radium-223 as a result of the recurrent anemia. A restaging bone scan and MRI were performed after the third cycle. The bone scan demonstrated a mixed response of the bone metastases with ongoing superscan appearance; however, heterogeneity of bone remodeling in the lower thoracic spine and distal left femur consistent with metastatic disease improvement was observed (Figure 5B). Unfortunately, a restaging MRI of the craniospinal axis demonstrated a local recurrence of the tumor in the posterior fossa without evidence of spinal metastases (Figure 6). The remaining three cycles of radium-223 were discontinued in light of the patient’s disease progression with recurrent soft tissue metastases. In terms of pain response, the patient noted minimal symptomatic relief and no significant reduction in opioid analgesic usage after three cycles of radium-223. The patient declined further palliative external beam radiotherapy to the local recurrence in the posterior fossa and also declined palliative chemotherapy. He received palliative care and died 8.7 months after the initiation of radium-223.

## Discussion

This case report is the first to describe the use of radium-223 in an adult patient with the rare presentation of recurrent extraneural metastatic medulloblastoma with diffuse bone-only metastases and a superscan on imaging. Mokhtech et al. reported long-term outcomes of adult patients with medulloblastoma and found that isolated bone metastases are more common in adults than children, with 36% of recurrences being bone metastases [5]. The authors recommended including positron emission tomography (PET) or bone scan on initial staging and during follow-up of adult patients with medulloblastoma. Diffuse bone metastases in adult patients with medulloblastoma with a superscan are very uncommon. Pathologic confirmation of recurrent metastatic disease is critical to rule out benign causes of a superscan or more common malignant histologies associated with a superscan.

The European Association of Neuro-Oncology and European Rare Cancer guidelines for the treatment of adult medulloblastoma state that standard treatment involves maximal safe resection, craniospinal radiation therapy, and chemotherapy. However, there is no definite recommendation for the treatment of relapse [6]. Genetic testing demonstrated an activating PIK3CA mutation, which is identified in approximately 3% of medulloblastomas; however, the predictive and prognostic significance of this mutation in medulloblastoma remains uncertain and did not impact the patient’s management options [7,8]. More importantly, in cases when the patient does not consent to systemic therapy, alternative treatment options are limited. The role of chemotherapy in medulloblastoma relapse has not been studied in adults, so treatment recommendations are generally extrapolated from the pediatric literature [6,9]. Although chemotherapy or other systemic therapies would be considered the primary treatment for patients with metastatic disease, the use of bone-directed radionuclide therapy for bone-only metastases could be a potential alternative treatment option in patients who decline or have contraindications to chemotherapy, extrapolating from its use in other malignancies where patients present with a superscan, such as prostate cancer [4].

This report highlights the use of radium-223, which is not well-studied in non-prostate cancer patients. Radium-223 mimics the uptake of calcium in osteoblastic lesions due to its similar chemical properties and involves the emission of high-energy alpha particles in the intralesional bone matrix. This leads to the induction of potentially cytotoxic DNA double-strand breaks in cells with a relatively short 100-micrometer distance while sparing surrounding normal tissue [10]. These physical characteristics generally result in a lower risk of myelosuppression than beta-emitting radioisotopes, such as strontium-89 or samarium-153 [11]. Radium-223 is approved for use in mCRPC based on a randomized phase III trial, which demonstrated improved overall survival and symptomatic control [4]; however, its effect in non-prostate cancer bone metastases is unclear, and to our knowledge, there are no published reports on its use in extraneural metastatic medulloblastoma. The mechanism of action for radium-223 is not histology-specific and could be effective in any patient with diffuse bone-only metastases with osteoblastic lesions on imaging [10]. The patient’s poor tolerability of radium-223 may have been impacted by the prior craniospinal irradiation affecting his bone marrow reserve.

There are few published studies on the utility of radium-223 in other cancer diagnoses. Ueno et al. conducted a phase II study on the efficacy and safety of radium-223 with hormonal therapy in hormone receptor-positive, bone-dominant metastatic breast cancer and reported a disease control rate of 49% at nine months, a tumor response rate of 54% at six months, and median progression-free survival (PFS) of 7.4 months [12]. There were no grade 3 or 4 adverse events reported, and the authors concluded that radium-223 has possible efficacy in hormone receptor-positive, bone-dominant metastatic breast cancer [12]. On the other hand, a phase II study by Deandreis et al. using radium-223 in radioactive iodine refractory bone metastasis from thyroid cancer was stopped after first-step analysis due to a lack of metabolic response [13]. Both of these phase II studies and this case report used the standard dose regimen of radium-223 for mCRPC. Given the discordant results of these two phase II studies, further research is needed to determine the patient populations that will derive the most benefit from expanded indications for radium-223. The definite impact of radium-223 on overall survival and palliation in non-prostate cancer patients remains to be established but is deserving of further study due to the large population of patients with bone metastases that could potentially benefit from this treatment.

## Conclusions

Recurrent extraneural metastatic medulloblastoma in an adult with diffuse bone-only disease and radiologic presentation with a superscan is very rare. In patients who decline or have contraindications to chemotherapy in this setting, radionuclide therapy with radium-223 may be explored as a potential alternative, especially in the absence of definitive evidence to guide further management. When doing so, blood work should be closely monitored for myelosuppression and managed appropriately. Further research on the use of radium-223 for patients with metastatic bone metastases from non-prostate cancer primary tumors is warranted.
